# Characterization of Mucosal-Associated Invariant T Cells in Oral Lichen Planus

**DOI:** 10.3390/ijms24021490

**Published:** 2023-01-12

**Authors:** Lara Marie DeAngelis, Nicola Cirillo, Alexis Perez-Gonzalez, Michael McCullough

**Affiliations:** 1 Melbourne Dental School, The University of Melbourne, Carlton, VIC 3053, Australia; 2 Melbourne Cytometry Platform, The University of Melbourne, Parkville, VIC 3010, Australia

**Keywords:** oral lichen planus, candidosis, mucosal immunity, mucosal-associated invariant T cell, mucocutaneous disorder, multiplex immunohistochemistry

## Abstract

Oral lichen planus (OLP) is an inflammatory condition of unknown cause that has been associated with concurrent candidal infection. Mucosal-associated invariant T (MAIT) cells express the T cell receptor TCRVα7.2 and are activated by riboflavin intermediates produced by microbes. The interaction between MAIT cells, *Candida*, and OLP is unknown. This study aimed to determine mucosal-associated T cell presence in OLP and whether the abundance of these cells changed due to the presence of either *Candida* or symptoms, using multiplex immunohistochemistry (mIHC). Ninety formalin fixed-paraffin-embedded (FFPE) tissue samples were assessed using mIHC for the cellular markers CD3, interleukin 18 receptor one (IL18R1), TCRVα7.2, CD161, CD8, and major histocompatibility complex class I-related (MR-1) protein. The samples were stratified into five groups on the basis of clinical (presence/absence of symptoms) and microbiological (presence/absence of *Candida*) criteria. Results demonstrated the presence of MAIT cell phenotypes in OLP inflammatory infiltrate within the connective tissue. Significant differences existed between different OLP groups with the percentage of log(CD3^+^ CD161^+^) and log(CD3^+^ TCRVα7.2^+^) positive cells (*p* < 0.001 and *p* = 0.005 respectively). Significant differences also existed with the relative abundance of triple-stained log(CD3^+^ CD161^+^ IL18R1^+^) cells (*p* = 0.004). A reduction in log(CD3^+^ CD161^+^ IL18R1^+^) cells was observed in lesional tissue of patients with symptomatic OLP with and without *Candida* when compared to controls. When present in OLP, MAIT cells were identified within the connective tissue. This study demonstrates that mIHC can be used to identify MAIT cell phenotypes in OLP. Reduced percentage of log(CD3^+^ CD161^+^ IL18R1^+^) cells seen in symptomatic OLP with and without *Candida* suggests a role for these cells in OLP pathogenesis.

## 1. Introduction

Oral lichen planus (OLP) is a chronic mucocutaneous condition of unknown cause that affects 1–2% of the population [1] presenting more frequently in women around the time of middle age. OLP is characterised by a persistent cytotoxic T cell inflammatory process directed against the basal keratinocytes and underlying basal lamina [2,3]. What triggers and maintains this inflammatory process is unknown. However, heat shock protein [4], hepatitis C virus [5,6], human papillomavirus [7], genetics, and stress [8,9] have been postulated as possible triggers.

Mucosal-associated invariant T (MAIT) cells are a unique population of T cells present in peripheral blood at rates of 1–10% [10,11,12]. MAIT cells can also be found in both the liver and gut mucosa [10,11]. These cells could play an active role in the aetiopathogenesis of OLP as the evolutionarily conserved invariant T cell receptor TCRVα7.2 can be activated by riboflavin intermediates that are bound to major histocompatibility complex class I-related (MR-1) protein [10,11,12]. Oral microbes, including *Candida*, may produce the riboflavin by-products required for MAIT cell activation. It has been shown that MAIT cells can be activated by epithelial cells infected with the invasive bacteria *Shingella flexneri*, with MAIT cells killing the infected cells expressing MR-1 [13]. Orally, this may be significant, as *Candida* spp. can superficially invade the epithelium, potentially triggering MR-1 production, MAIT cell activation, and subsequent keratinocyte death [14].

Evidence exists that the function and number of MAIT cells are altered in multiple diseases, including systemic lupus erythematosus (SLE), a condition that—when present in the oral cavity—clinically and histologically mimics OLP. In one example, MAIT cells were significantly reduced in SLE [15]. Furthermore, in the SLE cohort, MAIT cell deficiency was correlated with disease activity [15]. More recently, a further investigation of the role of MAIT cells in SLE also confirmed a reduction in MAIT cells in SLE patients that correlated with disease activity [16]. A recent pilot study assessing the function and characterisation of circulating MAIT and γδT cells in OLP demonstrated reduced frequencies of both cell types, alluding to a potential role in OLP pathogenesis [17].

MAIT cells express multiple surface markers and co-localisation of these markers can be used for identification. CD3, interleukin 18 receptor one (IL18R1), CD161, and TCRVα7.2 are markers that have been previously utilised to quantify MAIT cells in tissue [18,19,20,21]. However, concurrent staining with all these markers has never been attempted before and could help provide a more accurate characterization of MAIT cells and their association with disease states.

The aim of this study was to determine if MAIT cells are present in the oral mucosa of patients with OLP, and whether the number of MAIT cells was affected by the presence of *Candida* or symptoms. This study further aimed to quantify single-antibody and T cell phenotypes associated with MAIT cell markers.

## 2. Results

### 2.1. Patient Characteristics

Of the 90 formalin-fixed paraffin-embedded (FFPE) samples, one was excluded due to over-exposure, reducing the study population to 89 samples sourced from 88 patients, including 65 (74%) females and 23 (26%) males with a mean age of 61.1 years at the time of diagnosis, with no significant differences existing between the groups.

### 2.2. Optimization of Technique

**Figure 1:**
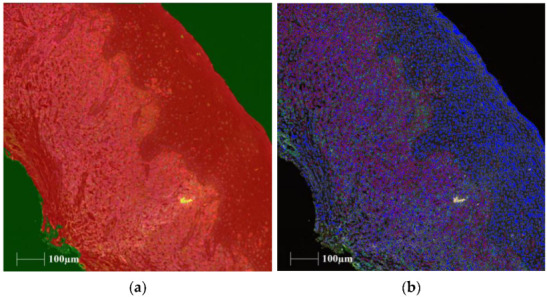
Representative images of trainable tissue segmentation and phenotyping performed with HALO™. (**a**) Tissue segmentation, (**b**) Phenotyping. Legend (**a**): red area = “tissue”, green area = “not tissue”, and yellow area = “tissue folds”. Legend (**b**): blue nucleus = DAPI positive, red membrane = CD3 positive, orange membrane = CD8 positive, cyan membrane = CD161 positive, yellow membrane = IL18R1 positive, magenta membrane = MR-1 positive, and green membrane = TCRVα7.2 positive.

We undertook a pilot experiment to determine optimal experimental conditions. For the tissue segmentation algorithm, tissue was defined into three categories: “tissue”—areas containing unfolded tissue with cells; “tissue folds”—areas where the tissue was folded over itself; and “not-tissue”—areas containing no tissue or cells. Bland–Altman plots performed on natural-log-+1-transformed data showed one comparison beyond the limits of agreement for “tissue” and “not tissue”. Percentage variability performed on raw data was low—less than 11% for all variables (Table 1). For the antibodies, Bland–Altman plots performed on natural-log-+1-transformed data showed comparisons beyond the limits of agreement for all antibodies except CD161. Analysis of variability between the two techniques using raw data revealed that all antibodies tested, except CD8, exhibited less variability with HALO™ compared to inForm (Table 1; representative HALO™ images are shown in Figure 1). Taken together, these preliminary results suggested that the HALO™ algorithm was more reliable, and hence, it was the algorithm chosen for all subsequent analyses.

### 2.3. Assessment of Single Antibody Phenotypes in OLP

We undertook co-localisation experiments of DAPI with CD3, CD8, CD161, MR-1, IL18R1, and TCRVα7.2 with ANOVA performed on natural-log-+1-transformed data.

Analyses with ANOVA identified significant differences between the groups between the number of log(CD3^+^)-, log(CD161^+^)-, and log(TCRVα7.2^+^)-positive cells (*p* = 0.017, *p* = 0.001, and *p* = 0.002, respectively). Tukey post hoc testing showed the OLP asymptomatic group exhibited significantly higher numbers of log(CD3^+^)-positive cells compared to the OLP *Candida* asymptomatic group (*p* = 0.024). Both OLP asymptomatic and OLP *Candida* asymptomatic groups exhibited significantly higher numbers of log(CD161^+^)-positive cells compared to the OLP symptomatic group (*p* = 0.003 and *p* = 0.004 respectively). Both OLP *Candida* asymptomatic and OLP *Candida* symptomatic groups exhibited significantly lower numbers of log(TCRVα7.2^+^)-positive cells compared to the OLP symptomatic group (*p* = 0.005 and *p* = 0.017, respectively). No other significant differences were noted.

### 2.4. Assessment of T Cell Phenotypes in OLP

T cell phenotypes were determined via co-localisation with DAPI plus CD3 with CD8, CD161, and IL18R1, with phenotypes reported as a percentage of the total CD3 population (Table 2). ANOVA was performed on natural-log-+1-transformed data.

Significant differences existed with regards to the percentage of log(CD3^+^ CD8^+^)-, log(CD3^+^ CD161^+^)-, and log(CD3^+^ TCRVα7.2^+^)- positive cells (*p* < 0.001, *p* < 0.001 and *p* = 0.004 respectively) on analysis with ANOVA. Tukey post hoc testing demonstrated both the OLP asymptomatic and OLP symptomatic groups showed significantly lower percentages of log(CD3^+^ CD8^+^) cells compared to the control group (*p* < 0.001 for both groups). CD8 staining was noted to be generally weak in OLP samples. The OLP-symptomatic group showed significantly lower percentages of log(CD3^+^ CD161^+^) cells compared to both the control and OLP-asymptomatic group (*p* = 0.001 and *p* < 0.001, respectively). Significant differences existed with regards to the percentage of log(CD3^+^ CD161^+^ IL18R1^+^)-positive cells (*p* = 0.004). Both OLP-symptomatic and OLP-*Candida*-symptomatic groups showed significantly lower percentages of log(CD3^+^ CD161^+^ IL18R1^+^)-positive cells compared to controls (*p* = 0.030 and *p* = 0.033, respectively). The OLP symptomatic group also showed a significantly lower percentage of log(CD3^+^ CD161^+^ IL18R1^+^)-positive cells compared to the OLP-asymptomatic group (*p* = 0.050). Taken together, these results indicated a reduction in the presence of log(CD3^+^ CD161^+^ IL18R1^+^) cells in lesional tissue of patients with OLP and who are symptomatic both when *Candida* is observed to be present in the tissue and when it is not.

### 2.5. Assessment of MAIT Cell Phenotypes in OLP

**Figure 2:**
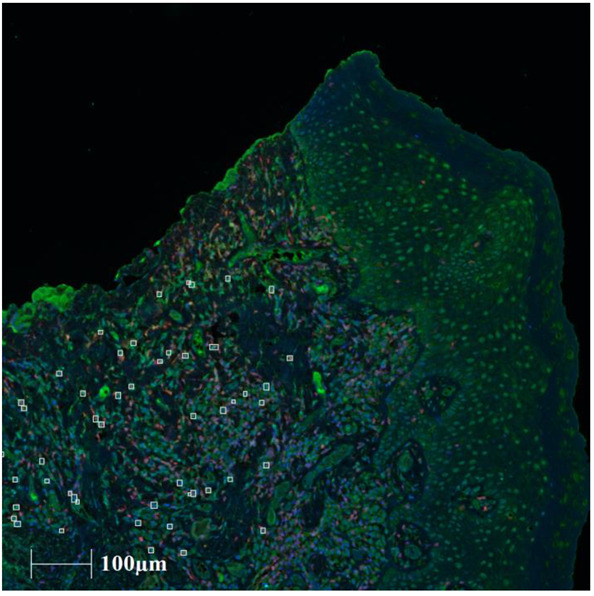
Representative image of MAIT cell phenotyping. OLP-asymptomatic sample after CD3^+^ CD161^+^TCRVα7.2^+^ phenotype analysis. Legend: White boxes identify the CD3^+^ CD161^+^ TCRVα7.2^+^-positive cells.

MAIT cell phenotypes, defined as a percentage of the total CD3 population expressing CD161, IL18R1, and/or TCRVα7.2, are shown in Table 3. The results show that MAIT cell phenotypes tended to congregate in the connective tissue where the majority of CD3 cells were located (Figure 2). Analysis with ANOVA showed significant differences between the groups for log(CD3^+^ TCRVα7.2^+^) (*p* = 0.005). Tukey post hoc testing demonstrated that the OLP-*Candida*-asymptomatic group exhibited significantly lower percentages of log(CD3^+^ TCRVα7.2^+^) cells when compared the OLP-symptomatic group (*p* = 0.006). No significant differences were noted with the other MAIT cell phenotypes.

## 3. Discussion

The present study assessed for the first time T cell and MAIT cell phenotypes in OLP patients with *Candida* and symptoms. We demonstrated the presence of MAIT cell phenotypes in OLP tissue, and to date, this is the first study to characterise MAIT cell phenotypes in OLP using mIHC.

Previous studies relied on double or triple staining of CD3, CD161, IL18R1 and TCRVα7.2^+^ to identify MAIT cells [18,19,20,21]. In the present study, multiple combinations of phenotypes were assessed to identify MAIT cells. We have demonstrated that MAIT cells tended to congregate within the connective tissue where the subepithelial lymphocytic infiltrate is located. A recent study has also shown the presence of MAIT cells in normal buccal mucosa in close proximity to the basement membrane [22] whilst a further characterised circulating MAIT and γδT cells in OLP using flow cytometry [17].

OLP is a T-cell-mediated, chronic, inflammatory condition of unknown cause, and the role that the oral microbiota may play in initiating a T cell response against oral keratinocytes has not been elucidated. Bacterial inflammatory-stimulated up-regulation of CXCL9/10 and over-representation observed in OLP suggests a role of innate immunity in OLP pathogenesis with microflora defence critical to this dysregulation [23,24]. The presence of *Candida* has been variably reported in 40–80% of OLP and 20–40% in controls [25,26]. It has been shown in an in vitro model that *C. albicans*, *C. glabrata*, and *Saccharomyces cerevisiae* have the ability to induce a MAIT cell response in an MR-1-dependent manner [27]. In the present study, the lack of variation observed between the OLP test groups and control with regards to MR-1 suggests that MR-1 expression was not a critical factor in determining variations in MAIT cell expression.

The presence of cytotoxic T cell phenotypes was found to be significantly lower in both the OLP-asymptomatic and symptomatic groups when compared to the control group, which was unexpected as this has been reported as the predominant inflammatory phenotype expressed in OLP [28]. However, this may be related in part to weak staining for CD8 throughout the present study. This is unlikely related to specimen storage or handling as the same sections were used for each antibody, thus eliminating antibody-specific variability due delay in fixation, fixative used, dehydration, drying, storage humidity, and temperature, which can all play an important role in the success of antigen retrieval [29,30]. The preservation of T lymphocyte surface membrane antigens in paraffin-embedded tissues has been previously shown to vary related to delays in fixation time, exposure to temperatures above 4 °C, and pH [31]. CD3 was shown to be the most stable, with CD8 being the most affected by sub-optimal processing and CD4 being intermediately affected [31]. Thus, a lack of robustness in the CD8 surface membrane with regards to variations in tissue fixation could account for decreased CD8 expression in the OLP cohort.

Of interest when investigating both single-antibody and MAIT cell phenotyping, the presence of *Candida* in OLP appeared have a significant decrease in log(TCRVα7.2^+^) cells and the percentage of log(CD3^+^ TCRVα7.2^+^) when compared to symptomatic OLP. This could suggest that the presence of *Candida* in OLP inhibits TCRVα7.2 expression in symptomatic OLP. Significant differences were also noted within the log(CD3^+^ CD161^+^ IL18R1^+^) phenotype, a T cell phenotype displaying key MAIT cell markers. Percentages of these cells were significantly lower in symptomatic OLP with and without *Candida* when compared to controls as well as in symptomatic OLP when compared to asymptomatic OLP. As with previous studies assessing MAIT cells in SLE and OLP [15,16,17], this study demonstrated reduced MAIT cell frequencies in OLP with specifically asymptomatic OLP with *Candida* when compared to symptomatic OLP, which could suggest a role for these cells in OLP pathogenesis.

In summary, CD3^+^ IL18R1^+^TCRVα7.2^+^, CD3^+^ CD161^+^ TCRVα7.2^+^, CD3^+^ CD161^+^ IL18R1^+^ TCRVα7.2^+^, and CD3^+^ TCRVα7.2^+^ are all phenotypes that could be used to identify MAIT cells. MAIT cells were shown to be present in OLP and when present congregated within the connective tissue. Lack of variation in the expression of MR-1 suggests no effect on determining variations in expression of MAIT cells within OLP or control groups. Identification of MAIT cells and reduced percentages of log(CD3^+^ CD161^+^ IL18R1^+^) cells in symptomatic OLP with and without *Candida* suggest a role for these cells in the pathogenesis of more active forms of OLP.

## 4. Materials and Methods

### 4.1. Patients

This retrospective analysis included 90 FFPE tissue blocks which were selected from 5 groups of patients, 30 samples of asymptomatic OLP, 30 samples of symptomatic OLP, 15 OLP samples with concurrent *Candida* present, (both asymptomatic (n = 6) and symptomatic (n = 9)), and 15 fibroepithelial polyp samples that served as controls. Samples were sourced from the Melbourne University Histopathology Service at the Royal Dental Hospital of Melbourne. These numbers are based on possible numbers of samples that were accessible for use as well as similar studies. One study by Hiejima, et al., 2015 [21], used a similar design and staining protocol for CD161 and TCRVα7.2 antibodies. Three study groups were included, with numbers of samples ranging from 10–15 samples per group. Another study by Li et al. [18] undertook a similar study with a similar staining protocol for CD3, IL-18Rα, and TCRVα7.2. This study included 5 study groups with 2–10 samples per group.

All samples were confirmed to be from oral mucosal tissue and were chosen based on a histopathological diagnosis of OLP, presence of *Candida* for the OLP with *Candida* cohort, and presence/absence of symptoms at the time of biopsy determined by the clinical description provided by the clinician on the biopsy report. Asymptomatic/minor activity OLP was defined as a report of no symptoms or very limited occasional discomfort. Symptomatic/high activity OLP was defined as OLP with reported ulceration and/or presence of ongoing symptoms on the biopsy report. This project was approved by the University of Melbourne Human Ethics Sub-Committee, project number 1749368.1.

### 4.2. Tissue Samples

4 µm thick sections were cut and mounted onto Superfrost^TM^ Plus (Thermo Fischer Scientific, Waltham, MA, USA) slides for subsequent periodic acid–Schiff (PAS) and mIHC staining. One sample in the OLP asymptomatic group (n = 29) was identified as having *Candida* with PAS staining and was reassigned to the OLP *Candida* asymptomatic group (n = 7).

### 4.3. Multiplex Immunohistochemistry

mIHC utilised the Opal^TM^ protocol. Automated dewaxing (Jung AutoStainer XL, Schalksmühle, Germany) was performed, followed by heat antigen retrieval with pH6 sodium citrate buffer using a pressure cooker. Endogenous peroxidase activity was quenched with 3% hydrogen peroxide. Protein blocking for 10 min was undertaken with tris-buffered saline and with bovine serum albumin with 0.25 M NaCl in a 1:1 ratio added to reduce ionic interactions. Blocking with avidin (DAKO, Jena, Germany) and biotin (DAKO, Jena, Germany) for 20 min each was also performed.

The first primary antibody was incubated for 1 h, followed by incubation of the secondary antibody for 30 min and fluorophore for 6 min. Heat antigen retrieval was performed again by placing the slides in pH6 sodium citrate buffer and heating for 2 min and 30 s in a microwave. After cooling, the cycle was repeated beginning with the application of the next primary antibody, and the process was repeated until all antibodies and fluorophores were applied with DAPI (1:1000) used for nuclear counterstaining.

Antibodies and fluorophores included TCRVα7.2 (Miltenyi Biotec, Bergisch Gladbach, Germany) 1:500, Opal 520 (PerkinElmer, Waltham, MA, USA); CD161 (Abcam, Cambridge, UK) 1:400, Opal 620 (PerkinElmer, Waltham, MA, USA); IL18R1 (Abcam, Cambridge, UK) 1:1500, Opal 570 (PerkinElmer, Waltham, MA, USA); MR-1 (Biorbyt, Cambridge, UK) 1:2000, Opal 650 (PerkinElmer, Waltham, MA, USA); CD8 (Abcam, Cambridge, UK) 1:500, Opal 540 (PerkinElmer, Waltham, MA, USA); and CD3 (Abcam, Cambridge, UK) 1:500, Opal 690 (PerkinElmer, Waltham, MA, USA).

TCRVα7.2, CD3, CD161, IL18R1 and MR-1 were chosen as they have previously been utilised for MAIT cell identification [18,19,20,21]. CD8 was chosen to quantify cytotoxic T cells, the predominant cells in the OLP chronic inflammatory infiltrate [28].

### 4.4. Quantitative Analysis and Phenotyping

Slide scanning was performed on all slides. Full-tissue multi-channel fluorescent scans were taken with the Vectra^®^ Automated Multispectral Imaging System (PerkinElmer, Waltham, MA, USA) at 10× magnification from 420 nm to 720 nm (excitation spectra) to generate one lower-power single-stack multispectral image (MSI) per slide [32]. Five random fields were chosen and imaged at 200× magnification using the stamp application in Phenochart (PerkinElmer, Waltham, MA, USA). These fields had a final resolution of 0.5 µm/pixel with an image size of 1338 µm × 1000 µm. These fields were scanned at high resolution with the Vectra^®^ Automated Multispectral Imaging System (PerkinElmer, Waltham, MA, USA) at 20 nm wavelength intervals [32]. These captures were combined to generate 5 high-powered single-stack MSI per slide. Component images were generated for 5 multispectral images (MSI) from each sample (total 445 images) using inForm. One sample from the OLP asymptomatic group was excluded from further analysis due to over-exposure (n = 89).

A pilot experiment to determine the most reliable method for phenotyping was undertaken on 20 OLP samples, 5 from each group, using both HALO™ and inForm software. Trainable tissue segmentation algorithms were created with HALO™ and inForm separating into “tissue”—areas containing unfolded tissue with cells; “tissue folds”—areas where the tissue was folded over itself; and “not-tissue”—areas containing no tissue or cells and applied to the 20 samples. Trainable single-antibody phenotyping algorithms with no co-localisation were also generated and applied to the 20 samples using HALO™ and inForm. HALO™ was the final chosen software, so all images were loaded into HALO™, and the 5 MSI per sample were merged, forming 89 merged images. An algorithm for tissue segmentation and phenotyping was created and applied to all samples using HALO™ to identify single antibody with co-localisation, T cell, and MAIT cell phenotypes.

### 4.5. Statistical Analysis

Natural log plus 1% (0.01) transformation was required to undertake statistical analysis with parametric tests. No transformation was required for the assessment of age, and chi-squared analyses were undertaken to assess gender related associations. Bland–Altman plots were used on transformed data to assess the agreement between the two digital methods of analyses, with analysis of the variability undertaken on the untransformed data by taking the standard deviation present across the 5 images of each sample and dividing by the sample’s mean value by the results expressed as a percentage. Mean and standard deviation between the test and control groups were presented using raw data. One-way analysis of variance (ANOVA) with Tukey’s post hoc testing undertaken on transformed data was used to assess the means of all groups. Analysis was performed using Mintab 18 and 19. Significance was defined as *p* value ≤ 0.05.

## Figures and Tables

**Table 1:** Percentage variability between tissue segmentation and single antibody analysis for HALO™ and inForm using raw data.

	HALO Variability (%)	inForm Variability (%)	Difference (%)
“Tissue”	33.8	23.5	−10.3
“Not Tissue”	52.0	55.2	3.2
“Tissue Folds”	99.1	93.1	−6.0
CD161	92.6	120.3	27.7
CD3	78.6	107.6	29.0
CD8	158.1	155.7	−2.4
IL18R1	109.4	141.9	32.4
MR-1	40.7	127.8	87.1
TCRVα7.2	143.7	166.4	22.7
DAPI	8.5	31.1	22.7

**Table 2:** Mean and standard deviation of T cell phenotypes as a percentage of the total CD3 population for the 5 groups presented using the raw data.

	CD3^+^ CD8^+^ µ ± SD (%)	CD3^+^ CD161^+^ µ ± SD (%)	CD3^+^ IL18R1^+^ µ ± SD (%)	CD3^+^ CD161^+^ IL18R1^+^ µ ± SD (%)
Control	5.3 ± 5.3	17.1 ± 15.2	24.1 ± 19.0	13.2 ± 14.8
OLP Asymptomatic	1.4 ± 3.5	14.3 ± 14.8	20.7 ± 19.1	9.5 ± 11.9
OLP Symptomatic	1.1 ± 2.8	5.7 ± 11.9	17.1 ± 24.5	5.1 ± 10.6
OLP *Candida* Asymptomatic	2.9 ± 6.6	10.3 ± 15.1	21.5 ± 25.9	8.3 ± 13.6
OLP *Candida* Symptomatic	3.4 ± 5.9	7.7 ± 8.6	12.7 ± 28.4	1.5 ± 3.5

µ represents the mean, and SD represents the standard deviation.

**Table 3:** Mean and standard deviation of MAIT phenotypes as a percentage of the total CD3 population for the 5 groups presented using the raw data.

	CD3^+^ TCRVα7.2^+^ µ ± SD (%)	CD3^+^ IL18R1^+^ TCRVα7.2^+^ µ ± SD (%)	CD3^+^ CD161^+^ TCRVα7.2^+^ µ ± SD (%)	CD3^+^ CD161^+^ IL18R1^+^ TCRVα7.2^+^ µ ± SD (%)
Control	8.2 ± 10.5	2.3 ± 4.2	2.3 ± 3.3	1.4 ± 2.4
OLP Asymptomatic	17.6 ± 26.0	4.1 ± 7.4	2.7 ± 4.9	2.0 ± 4.4
OLP Symptomatic	30.2 ± 33.6	7.0 ± 17.2	1.6 ± 3.6	1.5 ± 3.4
OLP *Candida* Asymptomatic	0.3 ± 0.6	0.1 ± 0.3	0.2 ± 0.4	0.1 ± 0.2
OLP *Candida* Symptomatic	13.9 ± 27.9	8.0 ± 23.9	3.4 ± 7.0	1.1 ± 3.3

µ represents the mean and SD represents the standard deviation.

## Data Availability

The data presented in this study are available on request from the corresponding author. The data are not publicly available due to privacy.
